# Botanical Ingredient Forensics: Detection of Attempts to Deceive Commonly Used Analytical Methods for Authenticating Herbal Dietary and Food Ingredients and Supplements

**DOI:** 10.1021/acs.jnatprod.2c00929

**Published:** 2023-01-30

**Authors:** Stefan Gafner, Mark Blumenthal, Steven Foster, John H. Cardellina, Ikhlas A. Khan, Roy Upton

**Affiliations:** † American Botanical Council, Austin, Texas 78714, United States; # Steven Foster Group, Eureka Springs, Arkansas 72632, United States; § ReevesGroup, Virginia Beach, Virginia 23451, United States; ▽ National Center for Natural Products Research, University of Mississippi, University, Mississippi 38677, United States; ∥ American Herbal Pharmacopoeia, Scotts Valley, California 95067, United States

## Abstract

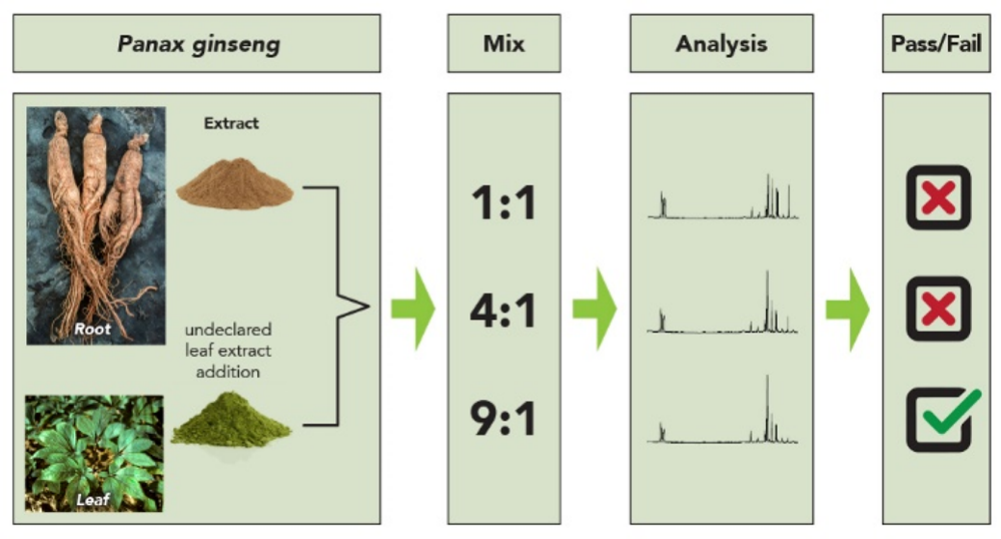

Botanical ingredients are used widely in phytomedicines, dietary/food supplements, functional foods, and cosmetics. Products containing botanical ingredients are popular among many consumers and, in the case of herbal medicines, health professionals worldwide. Government regulatory agencies have set standards (collectively referred to as current Good Manufacturing Practices, cGMPs) with which suppliers and manufacturers must comply. One of the basic requirements is the need to establish the proper identity of crude botanicals in whole, cut, or powdered form, as well as botanical extracts and essential oils. Despite the legal obligation to ensure their authenticity, published reports show that a portion of these botanical ingredients and products are adulterated. Most often, such adulteration is carried out for financial gain, where ingredients are intentionally substituted, diluted, or “fortified” with undisclosed lower-cost ingredients. While some of the adulteration is easily detected with simple laboratory assays, the adulterators frequently use sophisticated schemes to mimic the visual aspects and chemical composition of the labeled botanical ingredient in order to deceive the analytical methods that are used for authentication. This review surveys the commonly used approaches for botanical ingredient adulteration and discusses appropriate test methods for the detection of fraud based on publications by the ABC-AHP-NCNPR Botanical Adulterants Prevention Program, a large-scale international program to inform various stakeholders about ingredient and product adulteration. Botanical ingredients at risk of adulteration include, but are not limited to, the essential oils of lavender (*Lavandula angustifolia*, Lamiaceae), rose (*Rosa damascena*, Rosaceae), sandalwood (*Santalum album*, Santalaceae), and tea tree (*Melaleuca alternifolia*, Myrtaceae), plus the extracts of bilberry (*Vaccinium myrtillus*, Ericaceae) fruit, cranberry (*Vaccinium macrocarpon*, Ericaceae) fruit, elder (*Sambucus nigra*, Viburnaceae) berry, eleuthero (*Eleutherococcus senticosus*, Araliaceae) root, ginkgo (*Ginkgo biloba*, Ginkgoaceae) leaf, grape (*Vitis vinifera*, Vitaceae) seed, saw palmetto (*Serenoa repens*, Arecaceae) fruit, St. John’s wort (*Hypericum perforatum*, Hypericaceae) herb, and turmeric (*Curcuma longa*, Zingiberaceae) root/rhizome, among numerous others.

## Introduction

The use of medicinal plants as treatments for illness and/or as natural agents to maintain wellness has a long history, but along with the botanical trade followed the accidental or intentional sale of materials of lower value that are sometimes offered as if they were the desired ingredient.^1^ This adulteration results in variations in identity, strength, purity, and expected benefits or therapeutic outcomes from the claimed identity of a botanical ingredient. While the focus of this review is on botanical ingredients used for medicinal or nutritional purposes, the issue of adulteration goes beyond the herbal medicine and dietary/food supplement industry. It also applies to botanicals used in the food (in drinks, nutrition bars, etc.), personal care, and cosmetic industries.

For hundreds of years, organoleptic evaluations of an herbal drug, i.e., the assessment of the shape, color, odor, taste, or ability to break a root or bark, were the main way to establish the identity of botanical materials. However, as the botanical ingredient trade, especially in Western countries, has moved from crude botanicals to more concentrated forms such as extracts and essential oils, the establishment of the proper identity and authenticity of an ingredient or a finished product has become more challenging. At the same time, herbal medicines, dietary supplements, and food supplements have gained in popularity over the past few decades. According to sales data collected by the *Nutrition Business Journal*, global botanical dietary/food supplement sales are estimated to reach approximately US $45 billion in 2022, up from US $33 billion in 2017.^2^ The increase in demand, coupled with price increases and supply shortages for certain ingredients, has provided a fertile landscape for fraudsters to sell adulterated materials. Two articles reviewing the published literature on botanical ingredient and herbal product adulteration found the same percentage of adulterated materials (ca. 27%) independent of researchers using genetic or chromatographic and spectroscopic assays of authentication.^3,4^ The adulterated products usually represent a form of economic fraud, even if mistaken species identification, confusion of vernacular names, or permissible interchangeable use of plants can also be causes of incorrectly labeled products. Most types of adulteration do not constitute a safety risk, although there are some notable exceptions that will be discussed below. Additionally, adulteration with lower-cost ingredients puts reputable herbal dietary supplement manufacturing companies at a competitive disadvantage. The additional price pressure, in turn, can lead to more companies taking shortcuts in quality in order to sell their products.

Since its inception in 2011, the ABC-AHP-NCNPR Botanical Adulterants Prevention Program (BAPP), an independent consortium of nonprofit organizations consisting of the American Botanical Council (ABC), the American Herbal Pharmacopoeia (AHP), and the National Center for Natural Product Research (NCNPR) at the University of Mississippi, has published a series of articles and technical reports documenting adulteration and fraud for dozens of botanical ingredients. In most cases, adulteration is done by providing botanical ingredients that appear to comply with specifications and standardization requirements for specific marker/active compounds but, in fact, simply exploit the lack of specificity of the test method used to measure them. This overview details some of the most frequently used approaches to deceive common laboratory analytical methods.

The specific approach and amount of testing required to authenticate a botanical ingredient varies and may include organoleptic/macroscopic, microscopic, genetic, and chemical identification. Authentication methods for botanical materials are available in various official compendia (e.g., the *United States Pharmacopeia* [USP], the *European Pharmacopoeia* [EP], the *Chinese Pharmacopoeia* [PPRC]), as well as monographs from the unofficial American Herbal Pharmacopoeia (AHP) and Therapeutic Compendium. Such tests include UV/vis spectrophotometry (UV/vis), high-performance liquid chromatography (HPLC) with UV/vis, evaporative light scattering (ELSD) or refractive index (RI) detectors, and gas chromatography with flame ionization or mass spectrometric detection (GC-FID and GC-MS, respectively).

However, the fraudsters (a polite term; in many cases and jurisdictions they are regarded as criminals) are well aware of the commonly used identification assays and have developed schemes to foil such tests (Table 1). A good example to illustrate this can be found in the adulteration of so-called “grapefruit seed extracts” (presumably derived from the seeds of *Citrus* × *paradisi*, Rutaceae) and other allegedly natural materials, which are marketed as natural antiseptics or natural preservatives. Adulteration of commercial grapefruit seed products was first reported in 1991 with triclosan and methyl paraben as adulterants.^5^ Subsequent papers from the 1990s confirmed such practices but found benzethonium chloride as an additional adulterant.^6,7^ In the first decade of the present millennium, new adulterants such as benzalkonium chloride, cetrimonium bromide, and decyltrimethylammonium chloride were reported in commercial products labeled as “grapefruit seed extract”, likely representing an evolution in the adulteration scheme to evade detection by analytical methods that targeted triclosan, methyl paraben, and benzethonium chloride.^8^ The addition of cetrimonium bromide and decyltrimethylammonium, which lack a chromophore, can be seen as an effort to evade detection by HPLC-UV/vis systems, which are among the most commonly used analytical instruments in dietary supplement quality control laboratories. Though new analytical methods have improved the ability to establish the identity of plant-based ingredients and characterize their composition to aid in the detection of adulteration, unscrupulous ingredient suppliers and manufacturers have succeeded in finding ways to deceive potential buyers. In certain instances, as reported by some commercial laboratories, the fraudsters may even send a fake ingredient to a third-party contract analytical laboratory to determine if the adulteration can be detected.

**Table 1:** Adulteration Schemes for Important Medicinal Plant Extracts

Scientific Name	Common Name and Part	Analyte(s)	Adulterant	Methods at Risk of Being Deceived
*Actaea racemosa*	Black cohosh root	Triterpenoids	Other *Actaea* spp.	HPTLC, HPLC-UV/vis
*Crataegus* spp.	Hawthorn leaf and flower	Flavonoids	Rutin-rich extracts	HPTLC, HPLC-UV/vis, HPLC-MS, IR, NIR, Raman, NMR
*Curcuma longa*	Turmeric root	Whole extract	Undeclared excipients	HPTLC, HPLC-UV/vis, HPLC-MS
*Curcuma longa*	Turmeric root	Curcumin	Synthetic curcumin	HPTLC, HPLC-UV/vis, HPLC-MS, IR, NIR, Raman, NMR
*Echinacea angustifolia*	Narrow-leafed echinacea root	Echinacoside	*Cistanche* spp.	HPTLC, HPLC-UV/vis
*Echinacea* spp.	Echinacea herb and root	Whole extract	Undeclared excipients	HPTLC, HPLC-UV/vis, HPLC-MS
*Ginkgo biloba*	Ginkgo leaf	Flavonoids	Rutin-rich extracts	HPTLC, HPLC-UV/vis, HPLC-MS, IR, NIR, Raman, NMR
*Ginkgo biloba*	Ginkgo leaf	Whole extract	Undeclared excipients	HPTLC, HPLC-UV/vis, HPLC-MS
*Hydrastis canadensis*	Goldenseal root	Berberine	*Coptis* extract, Oregon grape extract, barberry extract	HPTLC, HPLC-UV/vis
*Panax ginseng*	Asian ginseng	Ginsenosides	Ginseng leaf extracts, *Panax quinquefolius* extract	HPTLC, HPLC-UV/vis, HPLC-MS
*Passiflora incarnata*	Passionflower herb	Flavonoids	Rutin-rich extracts	HPTLC, HPLC-UV/vis, HPLC-MS, IR, NIR, Raman, NMR
*Rhodiola rosea*	Rhodiola root	Cinnamyl alcohol glycosides, salidroside	Other *Rhodiola* species	HPTLC, HPLC-UV/vis
*Sambucus nigra*	European elder berry	Anthocyanins	Black rice extract	UV/vis, HPTLC, HPLC-UV/vis
*Serenoa repens*	Saw palmetto fruit	Fatty acids	Vegetable oils	HPTLC, GC-FID, GC-MS
*Silybum marianum*	Milk thistle fruit	Flavonolignans	Extracts from exhausted milk thistle seed	HPTLC
*Vaccinium macrocarpon*	Cranberry fruit	Anthocyanins	Black rice extract, hibiscus extract	UV/vis
*Vaccinium macrocarpon*	Cranberry fruit	Proanthocyanidins	Grape seed extract, peanut skin extract, pine bark extract	HPTLC, HPLC-UV/vis, IR, NIR, Raman, NMR
*Vaccinium myrtillus*	Bilberry fruit	Anthocyanins	Black rice extract, blueberry extract, mulberry extract	UV/vis

### Macroscopic Identification

Plant identification relies on the examination of specific taxonomic features in a plant and comparison of these features with other species. Plants in whole or cut form can be assessed using macroscopic identification, i.e., the evaluation of size, shape, color, texture, unique characteristics (e.g., annular rings on root material), or fracture (the manner in which a material breaks). One of the oldest ways to deceive macroscopic identification methods is the use of materials of similar shape or color.

There are numerous examples to illustrate this approach:

- the admixture of papaya (*Carica papaya*, Caricaceae) seeds to black pepper (*Piper nigrum*, Piperaceae) fruits;^9^
- the use of cut or pulverized *Cistus* spp. (Cistaceae) leaf, olive (*Olea europaea*, Oleaceae) leaf, thyme (*Thymus* spp., Lamiaceae) herb, sumac (*Rhus* spp., Anacardiaceae) leaf, hazelnut (*Corylus avellana*, Betulaceae) leaf, or myrtle (*Myrtus communis*, Myrtaceae) leaf as bulking materials for oregano (*Origanum vulgare*, Lamiaceae) leaf;^10^
- the substitution of arnica (*Arnica montana*, Asteraceae) flower with Mexican arnica (*Heterotheca inuloides*, Asteraceae) flower;^11^ or
- the sale of red-colored corn (*Zea mays*, Poaceae) stigmas, pomegranate (*Punica granatum*, Lythraceae) fruit peel, or pomegranate fruit fibers, red-dyed silk fibers, the stigmas from safflower (*Carthamus tinctorius*, Asteraceae), and calendula (*Calendula officinalis*, Asteraceae) flower as saffron (*Crocus sativus*, Iridaceae) stigmas.^12^

Lesser known examples include adulteration of nigella (*Nigella sativa*, Ranunculaceae) seeds with other seeds that are similar in size and color, such as black sesame (*Sesamum indicum*, Pedaliaceae), onion (*Allium cepa*, Liliaceae), or Mexican prickly poppy (*Argemone mexicana*, Papaveraceae),^13,14^ or the sale of berries from *Berberis* species (Berberidaceae) or *Sorbus* species (Rosaceae) labeled as sea buckthorn (*Hippophae rhamnoides*, syn. *Eleagnus rhamnoides*, Eleagnaceae) berries.^15^

In many cases, the adulterants are readily detected visually unaided or with the aid of a magnifying glass or a microscope, but in some instances, especially when bark or root material from closely related species are used as adulterants, orthogonal methods based on genetic or chromatographic/spectroscopic means may be needed to distinguish among species unequivocally.

Another way to deceive macroscopic identification is the sale of cut or powdered plant materials from which the valuable constituents have been removed. Such adulteration is known for spice plants such as black pepper,^16^ cinnamon (*Cinnamomum verum* and other *Cinnamomum* species, Lauraceae) bark,^17^ ginger (*Zingiber officinale*, Zingiberaceae),^18^ or paprika (*Capsicum annuum*, Solanaceae).^19^ This type of adulteration may be detected by organoleptic assessment, predominantly by the absence or changes in the expected color, texture, aroma, or taste, microscopically through the observation of ruptured cell walls indicative of pre-extraction, or quantitative analysis of the compounds of interest, e.g., using HPLC-UV/vis or GC-FID.

### Organoleptic Evaluation

Another important aspect of the initial authenticity evaluation is the organoleptic assessment, i.e., the taste, look, feel, and smell, of an herbal ingredient. While there are no good examples of intentional adulteration to mimic the determination of feel, imitations of look, taste, and smell are quite common, especially in powdered materials and extracts. A color similar to the labeled ingredient can be obtained by adding natural or synthetic dyes or by admixture or substitution of pigmented extracts with colorants from the same compound class. A well-known example is the adulteration of saffron with red-dyed corn (*Zea mays*, Poaceae) stigmas or other red-colored plant fibers and even paper or meat strips.^12,20^ Food dyes are sometimes also added to powdered plants to improve the visual aspects and impart a sense of higher quality. Examples for such adulteration are the addition of azo-dyes to paprika or saffron or the undeclared addition of lead chromate or Metanil Yellow to turmeric rhizomes.^21,22^ Although uncommon, the sale of bulk “bilberry” fruit extracts containing amaranth dye also falls within this category, although this type of adulteration is also meant to deceive assays determining the total anthocyanin content by UV/vis spectrophotometry.^23^ Also affected by this type of adulteration are fatty oils, where colorants are added to comply with organoleptic specifications. Examples are the undeclared addition of chlorophyll to vegetable oils labeled as olive oil^24^ or the addition of β-carotene to sunflower oil sold as sea buckthorn oil.^25^

Flavor imitation may be best known from vanilla, where synthetic vanillin is often sold as “natural vanilla” or “vanilla extract”.^26−29^ Such adulteration has become quite sophisticated as fraudsters, in some cases, enrich synthetic vanillin with ^13^C to obtain a ^13^C/^12^C isotope ratio (expressed as δ^13^C) value comparable to natural vanillin. However, specific stable isotope measurements can still detect such adulteration.^30^ Similar ways of flavor adulteration have been reported with cinnamon, wintergreen (*Gaultheria procumbens*, Ericaceae) herb, and birch (*Betula lenta*, Betulaceae) bark oils. In the case of cinnamon, the bark powder may be adulterated by, for example, preparing a mixture of powdered beechnut (*Fagus* spp., Fagaceae) husks that are aromatized with cinnamaldehyde. Cinnamon bark oil is commonly substituted with the less costly cinnamon leaf oil.^31^ Wintergreen and birch oils are known to be adulterated with synthetic methyl salicylate.^32−34^ This type of adulteration is most commonly detected using a number of stable isotope ratio (e.g., ^2^H/^1^H, ^13^C/^12^C, or ^18^O/^16^O) measurements.^32^

Adulteration of essential oils has a long history, since many of these oils are expensive and lower-cost ingredients, the addition of which is not easily detected, are readily available. Such adulteration is the most common way fraudulent operators are trying to deceive organoleptic tests for odor and aroma. Reviews on the topic have been written by Do et al.^35^ and Schmidt and Wanner,^36^ among others. Essential oils with a high adulteration risk are bergamot (*Citrus limon*, syn. *C. bergamia*, Rutaceae) peel oil,^35,37,38^ birch bark oil,^34^ cinnamon bark oil,^35^ Indian sandalwood bark oil,^35,39^ lavender flower oil,^35,37,38,40^ lemon balm (*Melissa officinalis*, Lamiaceae) leaf oil,^35,37^ peppermint (*Mentha* × *piperita*, Lamiaceae) leaf oil,^35,37^ rose flower petal oil,^35,37,41^ and tea tree leaf oil.^38,42^

A number of approaches are used to create an oil of a similar scent: (i) admixture or substitution with an essential oil of similar composition from a lower-cost material, (ii) a combination of essential oil fractions enriched in the essential oil constituents of interest, (iii) the creation of a blend of essential oil constituents made by chemical synthesis or biofermentation or obtained by fractionated distillation, or (iv) the steam-distillation of materials previously exposed to cold-pressing, especially peels from *Citrus* species.^36,43^ Often, the adulterating materials are added at concentrations that make the adulteration economically profitable but difficult to detect.

Although not often reported, the fragrance of botanical ingredients other than essential oils may be imitated by fraudsters. One such case was reported in India, where fake asafetida (*Ferula assa-fetida*, Apiaceae) powder was made with wheat (*Triticum* spp., Poaceae) flour to which asafetida water and a sulfur-containing material were added, giving it a typical asafetida smell.^44^

The most widely used means of detecting adulteration of essential oils is by gas chromatography. Often, adulterated essential oils do not comply with standards established by national or international pharmacopeias or the International Standardization Organization (ISO). In some cases, the use of a chiral stationary phase to determine the enantiomeric ratio of constituents of interest can be helpful, e.g., the ratios of (+)- and (−)-terpinen-4-ol, as well as (+)- and (−)-α-terpineol in tea tree oil. The enantiomeric ratios of these constituents are different in authentic tea tree oil from some of the adulterating materials.^45,46^ Similarly, the determination of the (+)-linalool/(−)-linalool and (+)-linalyl acetate/(−)-linalyl acetate ratios can be used to assess the authenticity of bergamot oil.^47^

In cases where synthetic constituents are used to adulterate the essential oil, byproducts of the synthesis may be detectable at low levels. In the case of lavender oil, byproducts from the chemical synthesis of linalool and linalyl acetate, e.g., dehydrolinalool, dihydrolinalool, dehydrolinalyl acetate, dihydrolinalyl acetate, plinol, and plinyl acetate, are indicators of adulteration with synthetically made compounds.^36,48^ For birch bark oil, the detection of dimethyl-2-hydroxyterephthalate is indicative of the presence of synthetic methyl salicylate.^34^ Application of mass spectrometry or specific natural-isotope fractionation nuclear magnetic resonance (SNIF-NMR) to determine stable isotope ratios has also been successfully applied, e.g., for the detection of adulteration of citrus oil,^49^ wintergreen oil,^32^ and oils from garlic, onion, and related plants (*Allium* spp., Amaryllidaceae).^50^

Additional methods that may help detect essential oil adulteration include high-performance thin-layer chromatography (HPTLC) or spectrometric or spectroscopic tests followed by multivariate statistical treatments such as principal component analysis (PCA), soft independent modeling of class analogy (SIMCA), and partial least-squares-discriminant analysis (PLS-DA).^35,51−53^

### Botanical Microscopy

The assessment of characteristic tissues in whole, cut, or powdered plant materials is still common in quality control laboratories. One of the most challenging tasks for a microscopist is to distinguish among closely related species due to the often-similar taxonomic features, which makes adulteration with closely related plants difficult to detect. There are many cases of intentional adulteration using such an approach, i.e., with plants from the same genus. Examples are the substitution of black cohosh (*Actaea racemosa*, Ranunculaceae), a plant that grows wild only in Eastern North America, with *Actaea* species of Asian origin;^54^ Asian ginseng (*Panax ginseng*, Araliaceae) with American ginseng (*P. quinquefolius*, Araliaceae);^55^ or eleuthero with other *Eleutherococcus* species.^56^ Similarly, rhodiola (*Rhodiola rosea*, Crassulaceae) is sometimes substituted with other *Rhodiola* species, although in this case, it may not be intentional, but rather due to confusion of vernacular names or permissible interchangeable use.^57^ To our knowledge, however, there are no examples where the adulterant was selected specifically to deceive authentication by botanical microscopy.

### Genetic Testing Methods

The use of DNA fingerprinting methods to authenticate plant species began in the 1980s,^58^ but the application of genetic approaches to authenticate dietary supplement ingredients gained significant attention only after 2010, when contract analytical laboratories started to offer authentication services based on DNA barcoding, and academic laboratories published findings on the composition of commercial herbal dietary supplements using DNA barcoding, DNA metabarcoding, high-resolution melting, shotgun sequencing, and other DNA-based methods.

Many genetic test methods are relatively easy to fool, i.e., by using a different part of the labeled plant (e.g., Asian ginseng leaf rather than root) or by adding inert materials (lactose, maltodextrin) that do not contain any DNA. Such adulteration has been reported, but its appearance prior to the advent of genetic testing of herbal dietary supplement products suggests that it was not done with the goal to deceive DNA testing. In practice, purposeful attempts to fool genetic methods are likely very rare, possibly because DNA-based identification is not currently a sufficiently common approach to determine species identity or product authenticity.

### UV/Vis Spectrophotometry

Mainly a quantitative method, ultraviolet/visible (UV/vis) spectrophotometry is widely used by botanical ingredient suppliers and dietary supplement manufacturers, and results from spectrophotometric assays are sometimes the sole data on the chemical composition of a botanical ingredient provided on a Certificate of Analysis (CoA) issued by an ingredient supplier. Advantages of spectrophotometry include the ease of use, high throughput (e.g., by using 96- or 368-well plates), and the comparatively low cost of the instrumentation. Applications are usually limited to compounds or compound classes that have an extensive chromophore and absorb visible light, typically in the 450–600 nm range, although some assays are carried out in the UV range (190–400 nm).

Marketers of dietary supplements may prefer results of spectrophotometric assays over more specific tests such as HPLC-UV/vis. This is because the concentrations obtained by spectrophotometry are often higher than the results from other quantitative methods due to interference from nontarget analytes that absorb at the wavelength of interest. A well-known example is silymarin, a flavonolignan mixture obtained from the fruits (often called “seeds”) of milk thistle (*Silybum marianum*, Asteraceae). Silymarin concentrations are generally between 30% and 65% by HPLC-UV and 65–80% using spectrophotometric tests.^59^ It should be emphasized that the declaration of UV/vis analytical results for silymarin on a certificate of analysis or commercial product label is not fraudulent per se, but declaration of methodology used to obtain the quantitative results should become standard industry practice.

The most common way that fraudsters are able to take advantage of the lack of specificity in UV/vis methods is the partial or full substitution with lower-cost extracts containing the same (or similar) type of constituents as the genuine extract. One such example is the adulteration of bilberry extracts with extracts from other anthocyanin-rich plants, e.g., blueberry species (*V. angustifolium*, *V. corymbosum*, *V. floribundum*, Ericaceae), wild cherry (*Prunus avium*, Rosaceae), black chokeberry (*Aronia melanocarpa*, Rosaceae), European elder berry, black soybean (*Glycine max*, Fabaceae) hull, black rice (*Oryza sativa*, Poaceae) fruit, mulberry (*Morus australis, M. nigra*, Moraceae) fruits, and others.^60−63^ While the usual costs of some of these fruit-derived materials are relatively high (e.g., compared to the always much lower costs of commodity ingredients like black soybean and black rice), these fruits are still available at lower cost in relation to bilberry.

The same approach is used in the adulteration of elder (*Sambucus* spp.) berry extracts. The commercial success during the COVID-19 pandemic and subsequent supply shortage and price increases of elder berry products has incentivized fraudulent operators to use the same approach and sell elder berry extracts diluted or substituted with black rice or purple carrot (*Daucus carota* var. *atrorubens*, Apiaceae) extracts.^64,65^

Adulteration with anthocyanin-rich extracts has also been reported for cranberry extracts. In a paper on the countercurrent chromatography (CCC) separation of anthocyanins from an extract labeled as cranberry, researchers successfully isolated delphinidin 3-*O*-sambubioside and cyanidin 3-*O*-sambubioside, two anthocyanins that do not occur in cranberry but are characteristic for hibiscus (*Hibiscus sabdariffa*, Malvaceae) extracts.^66^ Other adulterants falling into this category are black rice, black bean (*Phaseolus vulgaris*, Fabaceae), and mulberry extracts.^67,68^ A peculiarity of this type of adulteration is that the adulterant may itself be adulterated, depending on the market conditions, as shown by the example of elder berry. This goes to show that any botanical ingredient rich in anthocyanins may be subject to adulteration if there is sufficient profit to be made by the adulterator. In this regard, low-cost adulterants such as extracts of black rice, black soybean, and purple carrot may be more likely found as substitutes for more costly ingredients. Detection of such adulteration, however, is relatively easy using chromatographic techniques such as HPTLC or HPLC-UV/vis.^61,63,65^

A bit more challenging is the detection of adulteration in proanthocyanidin (PAC)-containing extracts. Separation of PACs, especially those with a degree of polymerization above 5, by chromatographic means is difficult; hence, suppliers generally use spectrophotometric methods for quantitative analysis. Since PACs do not have an extensive chromophore, these molecules need to be chemically modified to be measured in the visible range. Common derivatization agents are the Folin-Ciocalteau, vanillin-HCl, or 4-dimethylamino cinnamaldehyde (DMAC) reagents; another approach is the conversion of PACs into anthocyanins using the butanol-HCl assay.^69^ Since none of these spectrophotometric assays can distinguish among PACs from different sources, fraudsters use PAC-rich materials such as peanut (*Arachis hypogaea*, Fabaceae) skin, pine (*Pinus* spp., Pinaceae) bark, hibiscus calyx, and possibly other plants to fortify or substitute, e.g., cranberry^67,68^ and grape seed^70^ extracts. The detection of adulteration of PAC-rich extracts can be sometimes achieved using fingerprint methods, e.g., by HPTLC, HPLC-UV/vis or HPLC-MS, NMR, and MS,^71^ although chromatographic methods do not separate the larger PACs well and thus may not be suitable, depending on the ingredient. With regard to MS methods, matrix-assisted laser desorption/ionization time-of-flight (MALDI-TOF) MS has proven to be particularly useful to determine PAC fingerprints.^72^

The increasing popularity of mushroom-based dietary/food supplements, especially noticeable in the US market, has led to questions about the authenticity of mushroom supplements. Many of the marketed products are made from fungal mycelia grown on sterile rice or other grains or in liquid media containing a carbohydrate source.^73^ Chemical markers for the standardization of mushroom products are usually either polysaccharides or triterpenes. One of the commonly used ways to measure polysaccharide content is a spectrophotometric test using sulfuric acid and phenol as reagents.^74,75^ The sulfuric acid promotes polysaccharide hydrolysis and dehydration of the sugar, allowing a reaction with the phenol to form a colored product that can be measured at 490 nm.^76^ Since this method measures the sugar content of all types of mono-, oligo-, and polysaccharides, replacing the mushroom with lower-cost polysaccharides has been reported in the literature. Wu et al. documented the occurrence of starch in reishi (*Ganoderma lucidum*, Ganodermataceae) dietary supplements and noted that only five out of 19 commercial reishi dietary supplements complied with the label, while 13 of the products contained maltodextrin or other starch-like ingredients not originating from reishi. The authors employed a number of orthogonal methods, including HPTLC, GC-MS, saccharide mapping based on polysaccharide analysis using carbohydrate gel electrophoresis (PACE), and high-performance size-exclusion chromatography coupled with multiangle laser light scattering and refractive index detection (HPSEC-MALLS-RID), to determine the composition of these dietary supplements.^77^

A similar situation is the addition of food dyes to St. John’s wort extracts in order to obtain a numerical value for the absorption in the visible range that suggests that the extract contains hypericins at concentrations that comply with the labeled amount.^78,79^ Most often, a cocktail of four dyes, i.e., amaranth (FD&C Red #2), brilliant blue (FD&C Blue #1), sunset yellow (FD&C Yellow #6), and tartrazine (FD&C Yellow #5), is added. Brilliant blue and, to a much lesser extent, amaranth dye, absorbs light at 590 nm, the same wavelength where hypericin is measured. Some of the other dyes are presumably added to make the extract visually more similar to an actual St. John’s wort extract, so that the addition of brilliant blue is not readily observed by visual inspection.^80−82^ Adulteration of St. John’s wort extracts with food dyes can be detected, for example, using HPTLC or HPLC-UV/vis fingerprints.^78,79,81^

### Thin-Layer Chromatography and High-Performance Thin-Layer Chromatography

Thin-layer chromatography (TLC) and HPTLC are among the routine assays used for authentication of botanical ingredients and are usually part of the identification assays included in official pharmacopeias. HPTLC is currently the standard approach due to its superior resolution and reproducibility; it is a robust means to authenticate botanicals and to detect adulteration. Nevertheless, there are some cases, e.g., the genus *Euphrasia* (Orobanchaceae), where closely related species cannot be distinguished because of the intraspecific variability of constituents.^83^ Authenticity determination is also challenging when plants, such as *Euphrasia* species, easily hybridize.

Owing to its reliance on an entire chemical fingerprint for plant authentication, HPTLC is not easily deceived, although methods assessing solely the presence of one or several chemical markers, which may lack specificity, are still common. There are a few ways to fool such a method, most commonly the partial or entire substitution with extracts or purified fractions of a similar phytochemical composition. Examples of this include the addition of black rice extracts to elder berry extracts,^64,65^ the addition of Japanese sophora extracts to ginkgo extracts,^84−90^ the adulteration of black cohosh with *Actaea* species of Asian origin,^54^ or the adulteration of *Boswellia serrata* oleogum resin extracts with extracts from other *Boswellia* species.^91,92^

Many HPTLC methods may also be deceived by the addition of undeclared polar constituents, e.g., excipients like maltodextrin, maltose, lactose, or food dyes that do not migrate on silica gel plates and mobile phases with low polarity. One example is the addition of a food dye cocktail to St. John’s wort (detailed in the section on UV/vis Spectrophotometry). These polar constituents do not or barely migrate under conditions outlined in the *United States Pharmacopeia* (USP) or the *European Pharmacopoeia* (Ph. Eur.)^78^ and hence may be overlooked by the analyst assessing the chromatogram. However, such adulteration is easily detected using a modified mobile phase.^78^

Since HPTLC methods usually are not used in a quantitative manner, unethical suppliers or manufacturers may produce or sell excessively diluted botanical ingredients and products, respectively. Sometimes, adulterators may dilute with “spent” material, i.e., an ingredient that has part or all of the valuable constituents removed. Such ingredients have all the characteristic markers, and therefore may pass an identity test despite the low concentration present. These “weak” fingerprints have been noticed in a number of publications, e.g., with extracts made from milk thistle seed,^59,93^ ginkgo leaf,^88,94^ echinacea (*Echinacea angustifolia*, *E. pallida*, or *E. purpurea*, Asteraceae) root or herb,^95^ cranberry fruit,^96^ or St. John’s wort herb.^79^

One of the most challenging types of adulteration to detect is the fortification of a botanical extract with a pure or highly purified chemical marker. Examples are the undeclared addition of synthetic curcumin to turmeric root and rhizome extracts,^22,97^ rutin to ginkgo leaf extracts,^87^ and ellagic acid to pomegranate (*Punica granatum*, Lythraceae) peel extracts.^98,99^ The undeclared addition of chemical marker constituents from synthesis or fermentation is also a concern for many essential oils^35^ and will be described in more detail in the section on gas chromatography below.

The detection of chemical marker compounds obtained by purification from other plants or by chemical synthesis is very difficult. In some cases, byproducts from the isolation procedure (in the case of a marker from natural origin) or from the chemical synthesis can be used to detect the fraud. Girme et al.^100^ noticed the presence of (1*E*,4*Z*)-5-hydroxy-1-(4-hydroxy-3-methoxyphenyl)hexa-1,4-dien-3-one as a byproduct of the curcumin synthesis starting with acetylacetone (2,4-pentanedione) and vanillin. The authors detected the byproduct in four of 16 commercial turmeric samples purchased in India. Another approach to detect fortification with synthetic compounds is the determination of ^14^C concentrations by mass spectrometry, which can be used to distinguish natural from fossil-fuel-derived molecules. You et al.^97^ used this approach to determine the extent of biobased curcumin in “all-natural” turmeric dietary supplements sold in the USA. Five of the 14 turmeric dietary supplements were found to contain curcumin made using fossil-fuel-derived starting materials; thus, they were fortified with synthetic curcumin.

### Gas Chromatography with Flame Ionization or Mass Spectrometric Detection

Gas chromatography with detection systems such as mass spectrometers or flame ionization detectors (FIDs) is the method of choice to analyze volatile ingredients such as essential oils or supercritical CO_2_ extracts. Other applications include the determination of fatty acid contents, which is done by measuring the fatty acid methyl esters after acid-catalyzed conversion of the free and bound fatty acids, the measurement of fatty alcohols after derivatization with a silylation reagent, or the quantification of sterols.^82,101−106^

GC methods can be deceived by dilution of the labeled ingredient with nonvolatile materials, by addition of isolates/fractions similarly composed to the ingredient of interest, or by composing a product made from isolates that have a similar composition to the labeled botanical ingredient. The undeclared addition of natural or nature-identical compounds made by chemical synthesis or fermentation can go unnoticed if only a limited number of compounds are analyzed; this type of adulteration is particularly well-known in the essential oil industry. Examples include the addition of geraniol or geranyl acetate to rose essential oil,^35^ the spiking of lavender essential oils with linalool and linalyl acetate,^35,40^ the undeclared addition of citronellal or citral to lemon balm oil,^35^ and the fortification of iris (*Iris* × *germanica*, Iridaceae) oils with α-irone and β-irone.^35^ In certain cases, the marker compounds can also be added from lower-cost natural sources, such as the dilution of rose oil with palmarosa (*Cymbopogon martini*, Poaceae) oil,^35^ the undeclared addition of Ceylon citronella (*C. nardus*, Poaceae), Java citronella (*C. winterianus*, Poaceae), or lemongrass (*C. citratus*, Poaceae) to lemon balm oil,^35,36^ the substitution of lavender oil with lavandin (*Lavandula angustifolia* × *L. latifolia*, Lamiaceae) oil,^35,36,40^ or the addition of certain eucalyptus (*Eucalyptus globulus*, Myrtaceae) oil or pine oil fractions to tea tree oil.^35,42^ The use of GC columns that allow separation of chiral compounds, multidimensional GC, or the measurements of isotopic ratios or ^14^C contents can be used to detect some of these adulteration issues.^35,46,107^

Many of the more expensive seed oils used in culinary applications or as base ingredients in creams, lotions, and other cosmetic products have been subject to adulteration with lower-cost vegetable oils. Examples include olive oil,^24^ avocado oil,^108,109^ pomegranate seed oil,^110^ sea buckthorn oil,^25^ or nigella seed oil.^13,111^ Dilution or substitution of saw palmetto oil with palm (*Elaeis guineensis*, Arecaceae) oil, sunflower (*Helianthus annuus*, Asteraceae) oil, and coconut (*Cocos nucifera*, Arecaceae) oil is reportedly quite common in years when the saw palmetto harvest has been low.^112^ These oil mixtures often have a fatty acid profile that is indistinguishable from saw palmetto oil, but other parameters, such as color, odor, or contents of fatty alcohols or sterols may not comply with specifications for saw palmetto extract. A particularly sophisticated means to create “fake” saw palmetto oil has been reported by Perini et al.^113,114^ In this instance, fatty acids from animal sources were mixed with authentic saw palmetto oil to create an ingredient that complies with many of the specifications outlined in the USP.^105^

Several analytical methods have been described to detect adulteration of saw palmetto extracts.^115^ The color and/or odor of the adulterated material are often different from authentic saw palmetto, although color adjustments using carotenoids have been reported by industry members. Substitution or dilution with vegetable oils can be detected by the presence of unusually large amounts of certain fatty acids, e.g., linoleic acid and caprylic acid, and the acid value of saw palmetto is typically higher than values of other vegetable oils. More recently, chemometric methods for saw palmetto authentication have been proposed.^113,116,117^

### High-Performance Liquid Chromatography and Ultra-High-Performance Liquid Chromatography with UV/Vis or Mass Spectrometric Detection

**Figure 1:**
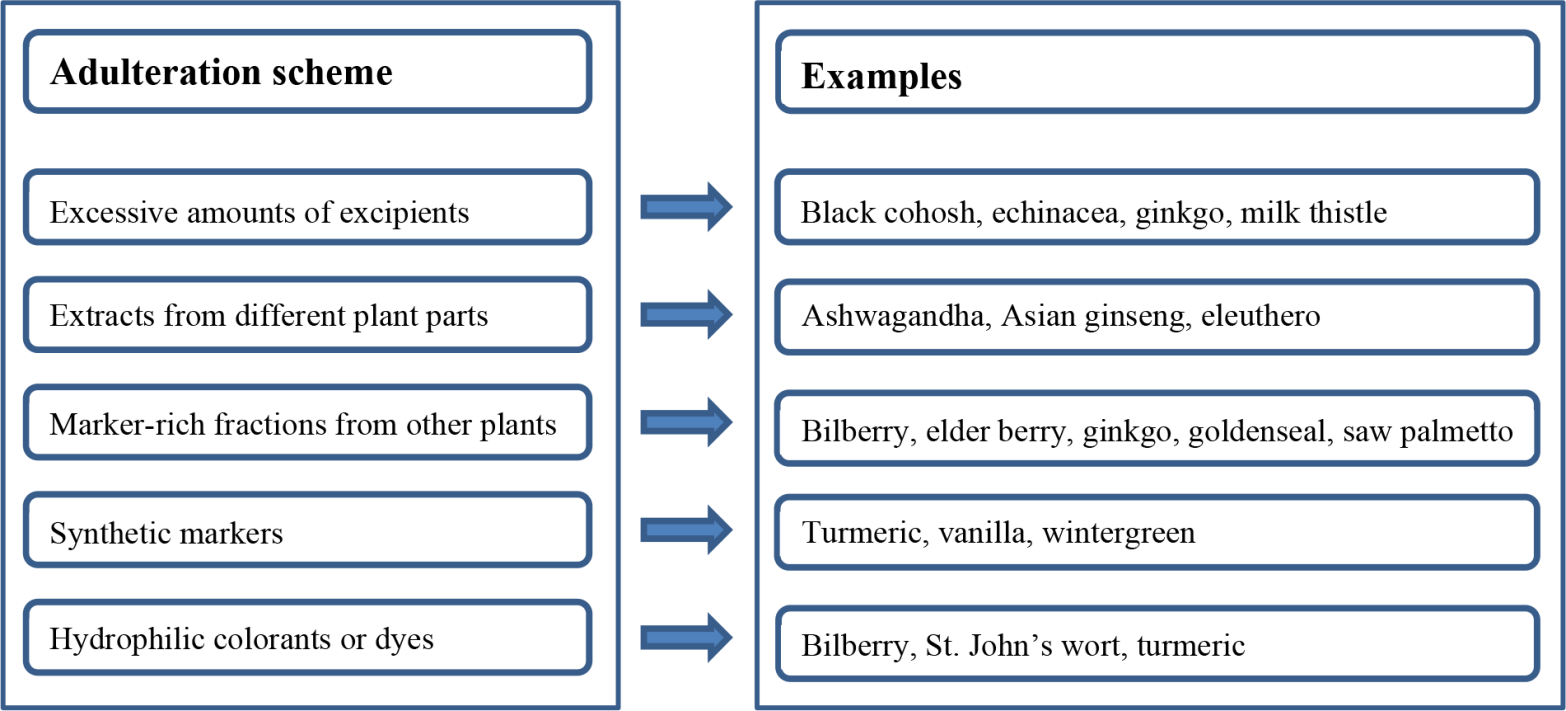
Examples of schemes to add lower-cost materials to botanical extracts, with the goal of going unnoticed by analysts using HPLC-UV/vis methods for authentication.

HPLC and ultra-high-performance liquid chromatography (UHPLC) are among the most widely used methods in dietary supplement quality control laboratories. They appeal due to their good separation capabilities and versatile detector options, which, depending on the detector setup, allow analysts to measure a large number of the molecules present in the extract. The most widely used detection system is the UV/vis detector, followed by the MS detector. While HPLC and UHPLC methods are more and more frequently used to assess the entirety of the detected molecules (“fingerprint”) using multivariate statistics, most pharmacopeial methods still rely on the presence/absence of one or several chemical marker compounds for authentication. Many of these chemical markers are phenolic compounds, which are readily measured by a UV/vis detector. However, these analytes are quite often molecules that are found in many plants and, therefore, are not suitable to identify a particular species. Commonly used schemes attempting to have the adulterating materials go unnoticed when analyzing botanical ingredients by HPLC-UV/vis are shown in Figure 1.

One way to obtain the desired marker compounds using a lower-cost material is the addition of extracts from the same plant but from a different plant part. Several authors^118−120^ reported adulteration of Asian ginseng (*Panax ginseng*, Araliaceae) root extracts with extracts of the leaves, which contain some of the same ginsenosides as the roots, although in different ratios.^120^ This type of adulteration has also been reported for sanchi ginseng (*Panax notoginseng*, Araliaceae).^121^ Another example is ashwagandha (*Withania somnifera*, Solanaceae) root. Thus, many of the withanolides found in ashwagandha root also occur in the aerial parts,^122^ a situation that has prompted unscrupulous suppliers to dilute or substitute root extracts with leaf extracts without indicating the presence of such extracts on the label.^123,124^ Similarly, extracts of eleuthero root may be adulterated with those of eleuthero aerial parts.^56^ The detection of such fraudulent activity is relatively straightforward if the entire chromatogram is considered. In the case of Asian ginseng, the relative concentrations of ginsenosides Rb1 and Rc are higher in the roots, whereas leaf extracts have higher relative amounts of ginsenosides Rd and Re. Ashwagandha leaves and stems have higher relative amounts of withaferin A, which can be used to detect adulteration with undeclared leaf extracts. Additionally, the aerial parts also contain flavonol glycosides that are absent in the roots.^123^ Since some of the adulterating parties are allegedly selling ashwagandha leaf extracts devoid of flavonoid glycosides, these constituents may not always be reliable chemical markers of adulteration. To the best of our knowledge, no clear distinction criteria for the various eleuthero plant parts have been established to date, since the chromatographic profiles of the roots, stems, and leaves appear quite similar.^125,126^ While complete substitution of root extracts with leaf extracts is not difficult to detect in most cases, the detection of admixtures of low amounts of leaf extract to root extract is challenging.

The most common mode of adulteration may be the use of chemical marker constituents from undeclared lower-cost plant sources. Extracts from berries rich in anthocyanins, such as bilberry or elder berry, are frequently adulterated with extracts from other anthocyanin-containing plants. Extracts of the roots of goldenseal (*Hydrastis canadensis*, Ranunculaceae), which are often standardized to contain a specific amount of the isoquinoline alkaloids berberine and hydrastine, may be subject to adulteration with other berberine-containing extracts, e.g., from coptis (*Coptis* spp., Ranunculaceae) roots.^127−129^ Apigenin purified from parsley (*Petroselinum crispum*, Apiaceae) can be used to adulterate extracts for which this flavonoid is used as a chemical marker, e.g., chamomile (*Matricaria recutita*, Asteraceae) extracts. Similarly, rutin-rich extracts or rutin has been used to adulterate ginkgo leaf extracts,^87^ passionflower (*Passiflora incarnata*, Passifloraceae) herb extracts,^130^ and chaste tree (*Vitex agnus-castus*, Vitaceae)^131^ or hawthorn (*Crataegus monogyna* or *C. laevigata*, Rosaceae) leaf and flower extracts.^131^ In most cases, a comparison of the chromatographic fingerprint with an extract made from botanically authenticated material is suitable to detect the fraud. The presence of constituents of unknown origin or unusually high contents of the chemical marker compared to the other plant metabolites are indicators of the addition of undeclared extraneous constituents/extracts. A particularly useful, although expensive setup is the combination of UHPLC with UV/vis, MS, and a charged aerosol detector (CAD), which provides qualitative and quantitative information on a majority of compounds in a given ingredient. This approach has been used successfully to distinguish among grape seed, peanut skin, and pine bark extracts.^132^

The adulteration of ginkgo leaf extracts with pure flavonols (rutin, quercetin) or flavonol-rich extracts has been reported in over two dozen studies.^133^ Compendial methods usually determine the flavonol glycoside content in ginkgo leaf extracts after acid hydrolysis in order to avoid quantification of the individual glycosides, which are difficult to separate and for many of which commercial standards are unavailable. The products of the hydrolysis are mainly quercetin, kaempferol, and isorhamnetin. The flavonols are more easily measured, but also make the method vulnerable to adulteration with extraneous flavonol-rich materials that have the same aglycones as the ginkgo leaves. In order to detect these types of adulteration, the USP has specified limits for rutin (not more than 4%) and quercetin (not more than 0.5%), measured prior to hydrolysis in the USP ginkgo leaf extract monograph.^134^ AHP recommends determination of the quercetin:kaempferol:isorhamnetin ratio after hydrolysis. In authentic ginkgo leaf extracts, the ratios range from 4:4:1 to 6:5:1. Significant deviation from these ratios is an indicator of potential spiking with a flavonol or a flavonol-rich extract from extraneous sources.^135^

As mentioned above, plant species or extracts where their identification is based on PACs represent a higher degree of difficulty due to the inability of chromatographic systems to separate the larger molecular weight PACs. Using reversed-phase chromatography, sufficient separation is limited to PACs with a degree of polymerization of 4 or less, while the rest of the polymers elute as broad, poorly shaped, overlapping peaks.^136^ When characteristic chromatographic fingerprint patterns for mono-, di-, tri-, and tetramers are lacking, it becomes almost impossible to distinguish among PAC-rich extracts by chromatographic means. Hence, expensive ingredients such as cranberry extract are sometimes partly or entirely substituted with lower-cost extracts from cranberry waste products or other plant sources. Such types of adulteration can easily go undetected if a manufacturer is relying solely on one or two monomers, e.g., catechin and epicatechin, for authentication of the ingredient.

Additionally, HPLC-based authenticity tests can be deceived by the addition of inert materials (sand, silica, maltodextrin), which are insoluble in widely used solvents such as methanol, ethanol, and aqueous mixtures thereof and hence may go unnoticed. Another possible issue is the undeclared addition of large amounts of excipients that lack a chromophore (e.g., sugars, sugar alcohols, and certain terpenoids) and therefore cannot be observed when a UV/vis detector is used.

### Infrared, Near-Infrared, and Raman Spectroscopy

Infrared (IR), near-infrared (NIR), and Raman spectroscopy are less widely used for assessing the authenticity of botanicals in the botanical dietary/food supplement industry, despite the relative low cost of the equipment and the fast sample preparation and analysis time. However, IR, NIR, and Raman spectroscopic procedures are used widely in the spice industry for the quality control of large shipments of raw plant material (e.g., leaves, roots, rhizomes, seeds) by developing a robust database from numerous authenticated plant samples. IR, NIR, and Raman spectroscopic methods are also quite frequently developed by researchers in academia to detect adulteration and to verify indications about the country/region of origin of vegetable oils such as olive oil.^103^ Assessments of authenticity are more easily done on cut or powdered plants than on extracts and finished products since processing differences, added excipients, and additional plants (in the case of the combination of ingredients in products) can lead to dramatic changes in the chemical composition and hence impact the ability to assess the similarity to a standard extract by multivariate statistical models. Specific schemes to avoid detection of adulteration by infrared and Raman spectroscopy are not known, although any attempt to evade detection is likely similar to those used for other chemometric methods, i.e., the admixture of plants or extracts with a similar chemical composition, or the fortification with purified marker constituents known to occur in the labeled botanical ingredient. Detection of echinacea root powder adulteration with roots from other *Echinacea* species or *Parthenium integrifolium* (Asteraceae), respectively, goldenseal root powder adulteration with roots of Oregon grape (*Berberis aquifolium*, Berberidaceae), yellow dock (*Rumex crispus*, Polygonaceae), yellow root (*Xanthorhiza simplicissima*, Ranunculaceae), and coptis (*Coptis chinensis* or *Coptis deltoidea*, Ranunculaceae), using NIR was achieved at adulterant concentrations of 5–15%.^137,138^ Walkowiak et al.^139^ assessed the usefulness of bidimensional FT-IR followed by data classification with multiway PCA (MPCA) to detect ginkgo leaf extract adulteration. Commercial ginkgo dietary supplement products fortified with rutin or kaempferol were readily detected. However, the separation of products fortified with either quercetin or a combination of rutin and a flavonol aglycone from authentic ginkgo leaf extract products was not achieved. Attempts to detect adulteration of commercial ginkgo products by NIR spectroscopy were unsuccessful due to the interference of excipients.^140^

### Mass Spectrometry

Mass spectrometry (MS) as a standalone approach and nuclear magnetic resonance (NMR, see below) have both been used mainly by research groups in academia to authenticate botanical ingredients and to detect adulteration. Authentication commonly is based on comparing MS fingerprints with botanically authenticated reference materials using multivariate statistical analysis. MS provides excellent results when comparing crude whole, cut, or powdered plants or batches of extracts that have been processed in a similar manner. Due to the sensitivity of MS detectors, adulterants can be traced at low concentrations.

A number of different MS variants have been employed for the authentication of olive oil: these include direct analysis in real time–time-of-flight (DART-TOF) MS, flow injection analysis–magnetic resonance mass spectrometry (FIA-MRMS), headspace MS, or electrospray–triple quadrupole (ESI-QQQ) MS.^103^ Depending on the approach, adulterations with other vegetable oils at concentrations of 1% could be detected, although a detection level of 5% or higher is more common. Other applications of direct MS are the authentication of black cohosh,^141^ cranberry,^142^ and St. John’s wort^143^ or the detection of skullcap (*Scutellaria lateriflora*, Lamiaceae) adulteration with species of the genus *Teucrium* (Lamiaceae).^144^

MS methods may be deceived by the fortification of extracts with purified single compounds or compound mixtures that occur in an ingredient of interest. Instances of such adulteration have been described in the TLC/HPTLC and HPLC sections of this review. Another potential issue is the presence of adulterants of low (<150 Da) or high (>1500 Da) molecular weights that may be outside the scan range of the experiments carried out with the mass spectrometers. However, we are unaware of any specific cases where fraudulent operators purposefully added adulterants with the goal of deceiving direct MS methods. This may be because direct MS is rarely used in a quality control setting of either a dietary supplement manufacturer or botanical ingredient supplier.

### Nuclear Magnetic Resonance Spectroscopy

Similar to MS, NMR-based methods for botanical ingredient authentication are most often based on a comparison of the NMR fingerprint with authentic reference standards using chemometric methods. This approach has been proven useful in the detection of adulteration of commercial extracts, although the use of different manufacturing processes, particularly the presence of excipients, can make data interpretation difficult.^79,140,141,145,146^ Other publications have focused on a more narrow part of the NMR spectrum: an example is the adulteration of saw palmetto, which can be detected using the proton signals of the glycerol moiety of triglycerides at ca. 4.20 and 5.25 ppm. These signals have a much lower intensity in saw palmetto than in some of its common adulterants.^116,118^

The undeclared addition of highly purified constituents from natural or synthetic sources is likely not be detected by NMR experiments, although some very specific NMR approaches have been developed, e.g., to detect the presence of synthetic vanillin in vanilla extracts.^147,148^ Other indications of the presence of fortified botanical extracts are the absence or low concentrations of important constituents, e.g., demethoxycurcumin and bisdemethoxycurcumin in turmeric extracts, or the presence of impurities from the synthesis of the curcuminoids.^149^ Another potential way to deceive NMR methods is the addition of undeclared high-molecular-weight PAC extracts, e.g., as adulterants to cranberry or grape seed extracts. NMR spectra of larger PACs (usually DP of 4 and higher) exhibit severe peak broadening in the spectrum at room temperature due to the rotational isomerism, necessitating low experimental (at ca. −20 °C) temperatures to obtain spectra that can be interpreted.^150,151^

### Safety Considerations

Since the adulteration schemes discussed here often involve addition or substitution with closely related plants from the same genus, or extracts containing the same chemical constituents/class of chemical constituents that are found in the labeled botanical, the risk of negative adverse health events due to the adulterant is low. The greatest safety concern regarding adulteration is the sale of undeclared pharmaceutical drugs marketed as dietary supplements, most notably in the erectile dysfunction, weight loss, and bodybuilding categories.^152^ Since the goal of this type of adulteration is to provide conventional drug-like benefits and not to deceive commonly used analytical methods, this is considered beyond the scope of the present contribution. However, included in this review article is the sale of synthetic industrial disinfectants such as benzalkonium chloride, benzethonium chloride, or triclosan labeled as “grapefruit seed extract”, since this represents a good example on how the approach to adulteration has changed over the years in attempts to evade detection. Frequent triclosan use has been linked to endocrine disruption, although clinical relevance is a matter of debate.^153,154^ Triclosan is also believed to increase the risk of antibiotic resistance, which is one of the reasons the ingredient was banned for use in antiseptic washes by the U.S. FDA in 2016.^154^ Benzalkonium chloride and benzethonium chloride have been used at low concentrations (less than 1 mg) in lozenges for sore throat, and a safety assessment by the European Medicine Agency for benzalkonium chloride listed “local irritation” as the primary concern.^155^ However, concentrations reported in some of the quaternary ammonium salt-containing “grapefruit seed extracts”, particularly of benzethonium chloride, were up to 50 mg/tablet, much higher than what has been used in pharmaceutical products.^156^ No safety data information on acute or chronic exposure to such high doses of benzethonium chloride could be retrieved.

Another safety concern is the addition of undeclared food dyes to herbal extracts such as the mixture of Amaranth, Brilliant Blue, Sunset Yellow, and Tartrazine to St. John’s wort herb extracts;^78^ Red II R, Rocceline, and Orange II to cinnamon bark extracts;^157^ Amaranth dye to bilberry fruit extracts;^23^ and Metanil Yellow, Sudan I, Sudan IV, or lead chromate to turmeric root extracts.^158^ Among those, Sudan I and Sudan IV are considered genotoxic and carcinogenic, and Sunset Yellow and Tartrazine have been linked to hyperactivity in children, although the causality is still a matter of debate.^159^ Of great concern is the use of lead chromate to improve visual aspects of turmeric roots, mainly in lower income areas in India and Bangladesh. Studies in Bangladesh indicate that a large portion of children have elevated blood lead levels, which, according to an investigation using the isotopic composition of lead, is due mainly to ingestion of foods prepared using turmeric powder containing undeclared lead chromate.^160^ High blood lead concentrations are known to be associated with impaired cognitive function.^161^

Finally, a known safety problem is the adulteration of skullcap, mainly with wall germander (*Teucrium chamaedrys*). Wall germander and other *Teucrium* species contain hepatotoxic neo-clerodane diterpenes that can cause acute liver injury.^162,163^ So while most types of adulteration do not pose a health risk, there are instances where the adulterant can lead to serious adverse events. However, there may be an indirect health risk for people using diluted and/or otherwise adulterated botanical ingredients in botanical dietary supplements and herbal medicines, since these products will likely not provide the health-related benefit that the consumer and/or health professional expected due to the lack of efficacy obtained.

## Conclusion

Due to the increasing demand worldwide for botanical raw materials, rising prices due to supply delays and shortages and inflation, and pressure on dietary and food supplement manufacturers to manufacture products at competitive prices, economically motivated adulteration is likely to remain an ongoing issue in the herbal medicine and dietary supplement industries, as well as the food, personal care, and cosmetic industries. Therefore, suppliers and manufacturers need to be aware of potential adulteration risks and ways that unscrupulous ingredient sellers attempt to deceive currently employed laboratory analytical methods. They also should establish appropriate, fit-for-purpose quality control measures to authenticate their ingredients. It is crucial that the quality control methods in use are specific enough to authenticate the botanical ingredient and to detect the adulterant. Since several types of adulteration may be observed for a single botanical ingredient, there is no standard quality control approach that can be used to detect all types of fraud. Ideally, for botanical ingredients that are confirmed as being subjected to adulteration, the use of an orthogonal testing protocol, which includes multiple complementary analytical methods, is warranted.
